# Early intravenous immunoglobulin use improves live birth outcomes in women with recurrent pregnancy loss: a propensity score–matched cohort study

**DOI:** 10.3389/fimmu.2026.1689166

**Published:** 2026-01-28

**Authors:** Wenhu Xin, Fangxiang Mu, Kexin Wang, Fang Wang

**Affiliations:** Department of Reproductive Medicine, Lanzhou University Second Hospital, Lanzhou, China

**Keywords:** intravenous immunoglobulin, live birth, pregnancy outcomes, propensity score matching, recurrent pregnancy loss

## Abstract

**Objective:**

This study aimed to evaluate the association between intravenous immunoglobulin (IVIG) treatment and pregnancy outcomes among women with recurrent pregnancy loss (RPL) in China.

**Methods:**

We conducted a retrospective cohort study involving RPL pregnant women who delivered at the Lanzhou University Second Hospital between April 2023 and August 2024. Participants were categorized into a treatment group (received IVIG during pregnancy) and a control group (not received). The pregnancy outcomes were live birth rate (LBR), preterm birth, birth weight, and neonatal unit admission. RPL pregnant women exposed to IVIG were matched to unexposed in a 1:1 ratio with propensity score matching (PSM), using the nearest neighbor matching. Multivariable logistics regression was used to assess the association between IVIG use during pregnancy and pregnancy outcomes. We further conducted a stratified analysis based on the mean daily dose and the gestational age at the initiation of IVIG administration.

**Results:**

A total of 504 RPL pregnant women were included, of whom 173 received IVIG during pregnancy and 331 did not. After PSM, 276 patients were analyzed with balanced baseline characteristics. The primary analysis showed that IVIG treatment during pregnancy was associated with a significantly higher LBR compared to controls (60.1% vs. 44.9%; adjusted OR [aOR]=1.960). This association remained significant after excluding cases with embryonic abnormal karyotypes (64.3% vs. 46.3%; aOR=2.187). Stratified analyses indicated that a mean daily IVIG dose <20 g was associated with improved LBR (aOR=2.484), and the benefit persisted after excluding abnormal karyotypes (aOR=3.000). Additionally, initiation of IVIG between 6–12 weeks’ gestation yielded higher LBR (72.8% vs. 44.9%; aOR=3.253), especially among participants without abnormal karyotypes (76.6% vs. 46.3%; aOR=3.757). No significant associations were observed between IVIG use and preterm birth rate, birth weight, or neonatal unit admission rate.

**Conclusions:**

IVIG use during pregnancy was associated with a significantly higher LBR among RPL women, particularly when initiated between 6–12 weeks of gestation and administered at a dose <20 g/d; these associations remained robust after excluding cases with abnormal karyotypes. These findings suggest that IVIG may be an effective immunological intervention for improving pregnancy outcomes in selected RPL patients.

## 1 Introduction

Recurrent pregnancy loss (RPL), commonly defined as two or more pregnancy losses, remains a distressing reproductive challenge affecting up to 5% of childbearing-age couples, and imposes a heavy emotional and physical burden. Although numerous causes have been proposed, including anatomical, genetic, endocrine, and immunological factors, nearly half of RPL cases remain unexplained (1). Currently, there is no proven effective therapy for RPL, and managing these patients remains difficult in clinical practice (1).

Immune dysregulation has been proposed as a potential contributor to RPL, involving altered maternal-fetal immune tolerance, increased natural killer (NK) cell cytotoxicity, a shift toward T helper 1 (Th1)-type inflammatory responses, and distributed cytokine networks (2). Intravenous immunoglobulin (IVIG) has been empirically used in some centers due to its immunomodulatory properties, such as Fc receptor blockade, modulation of complement activation, regulation of T cell subsets and cytokine profiles, and potential reduction of NK cell levels and cytotoxicity (3–5). However, the clinical effectiveness of IVIG in RPL remains inconclusive (6–8).

Existing evidence from randomized controlled trials (RCTs) meta-analyses has not demonstrated benefit of IVIG on live birth rates (LBR) in women with RPL. A recent systematic review and network meta-analysis of therapeutic interventions for idiopathic RPL reported that none of the evaluated interventions, including IVIG, resulted in a clear improvement in LBR or reduction in pregnancy loss rate compared with control (9). Similarly, an updated meta-analysis of RCTs including 10 trials found no statistically significant overall improvement in LBR with IVIG versus placebo; nevertheless, the authors reported that treatment effect appeared to increase with a higher number of previous losses (10). In addition, a randomized, double-blind, placebo-controlled trial conducted in women with unexplained RPL following assisted reproductive technology (ART) failed to demonstrate improved pregnancy outcomes with IVIG-based immunotherapy (11). Nevertheless, previous systematic reviews have highlighted considerable heterogeneity among IVIG studies with respect to patient selection, dosing regimens, timing of administration, and concomitant therapies (12). Moreover, several non-randomized and observational studies conducted in real-world settings have reported higher LBRs among IVIG-exposed women with RPL, further highlighting the potential heterogeneity in treatment response (13–15).

In recent years, the use of real-world data and robust statistical methods has become increasingly important in evaluating treatment effects, particularly in settings where RCTs are limited or infeasible. Although observational studies are more susceptible to bias than RCTs, well-designed studies can also provide valuable insights into real-world treatment outcomes (16). Propensity score matching (PSM) is a complementary tool to mimic randomized study characteristics partly (17), achieve balance comparability (18), and control for confounding bias in estimating treatment effects (19). It provides an appropriate methodology for assessing interventions in settings where RCTs are infeasible or limited.

Therefore, the present study aimed to investigate the association between IVIG use during early pregnancy and pregnancy outcomes among women with RPL using a PSM-based retrospective cohort design. In addition, we sought to explore whether the timing and dosage of IVIG administration influenced pregnancy outcomes. By utilizing real-world data, this study aims to provide clinically relevant evidence to inform safer and more effective use of IVIG in women with RPL.

## 2 Materials and methods

### 2.1 Study design

A retrospective cohort study was performed utilizing data collected from the RPL center at the Lanzhou University Second Hospital during the period from April 2023 to August 2024. The study enrolled pregnant RPL women who met the following criteria: (1) those who were 18 years of age or older; (2) those who had a record of delivery or termination of pregnancy in our hospital; (3) those who had a history of at least two pregnancy losses; and (4) those who had records on co-medication during pregnancy. The patients with incomplete information and who used teratogenic drugs during pregnancy were excluded from the study.

All the patients participating in the study received low molecular weight heparin (LMWH) as part of their treatment protocol. These patients were divided into two distinct groups based on their exposure to IVIG treatment during their pregnancies. The treatment group comprised pregnant RPL women who had received IVIG during their pregnancy, regardless of the specific dose and duration of the treatment. The control group included pregnant RPL women who had not received any IVIG treatment throughout their pregnancy.

At our center, IVIG dosing was primarily determined based on body weight. Patients weighing 40–55 kg typically received 15 g/d (2.5 g/vials × 6 vials), whereas those weighing 55–65 kg received 20 g/d (2.5 g/vials × 8 vials). All patients in the IVIG group started treatment during the first trimester of pregnancy, with the median gestational age at first administration of 6.35 weeks (range 2.28–12.14 weeks), and patients received a median of 2 administrations (range 1–11). Based on the treatment strategy in the first trimester of pregnancy, the treatment group received IVIG combined with LMWH, and the control group received LMWH only. During the second and third trimesters of pregnancy, the dose of IVIG was adjusted according to individual conditions.

### 2.2 Ethics approval

Approval for the study was granted by the Ethics Committee at the Lanzhou University Second Hospital (Reference No. 2019A-231), and all patients provided their written informed consent.

### 2.3 Data collection and outcome measures

Demographic information, medication data, and pregnancy outcomes were extracted from the medical record database. The drug information collected included: IVIG (dose; gestational weeks of the initial medication) and co-medications during pregnancy (prednisone, hydroxychloroquine, aspirin, ciclosporin, recombinant human granulocyte colony-stimulating factor, and tumor necrosis factor inhibitors). Pregnancy outcomes included LBR, preterm birth (<37 weeks’ gestation) rate, birth weight, and neonatal unit admission.

### 2.4 Confounding factors

Factors that could confound results were adjusted for by considering pre-pregnancy body mass index (BMI) (Underweight, Normal Weight, Overweight, and Obese), maternal age, number of previous pregnancy losses (1=“2”, 2=“≥3”), type of previous pregnancy loss (0=“Primary”, 1=“Secondary”), and co-medication during pregnancy (0=“No”, 1=“Yes”).

### 2.5 Statistical analysis

Descriptive analyses were performed to outline the characteristics of pregnant women with RPL who were receiving treatment with IVIG, as well as those in the control group. To assess the normality of continuous variables, the Kolmogorov-Smirnov test was utilized. For continuous variables that demonstrated a normal distribution, the results were reported as mean ± standard deviation (SD). Conversely, for those continuous variables that did not follow a normal distribution, the median and interquartile range (IQR) were utilized to present the data. Categorical data were presented as frequencies along with their corresponding percentages. To compare continuous variables, either the parametric Student’s *t*-test or the non-parametric Mann-Whitney test was employed. Categorical variables were compared using the chi-square test or Fisher’s exact test.

To estimate the effect of group accounting for confounding variables, a propensity score was utilized. This score was derived by applying a logistic regression model that included factors such as maternal age, pre-pregnancy BMI, number of previous pregnancy losses, types of previous pregnancy loss, and medications taken during pregnancy. Pregnant RPL women who were treated with IVIG were matched to those who were not treated in a 1:1 ratio using PSM, specifically employing the nearest neighbor matching method.

We assessed the relationship between IVIG treatment and pregnancy outcomes through logistic regression, calculating odds ratios (OR) and 95% confidence intervals (CIs). Initially, we analyzed the differences between the IVIG treatment group and the control group to explore the connection between drug exposure and pregnancy outcomes. Following that, a stratified analysis was performed by categorizing participants according to the mean daily dose and gestational weeks at which IVIG was first administered. Additionally, subgroup analyses were conducted stratifying participants by conception method (natural conception vs. ART) to examine whether the association between IVIG treatment and live birth differed between these groups. Interaction terms between IVIG treatment and conception method were included in logistic regression models to test for effect modification. *P* < 0.05 were considered statistically significant, and all tests were two-sided. The statistical analysis was carried out using SPSS software (version 25.0).

## 3 Results

**Figure 1:**
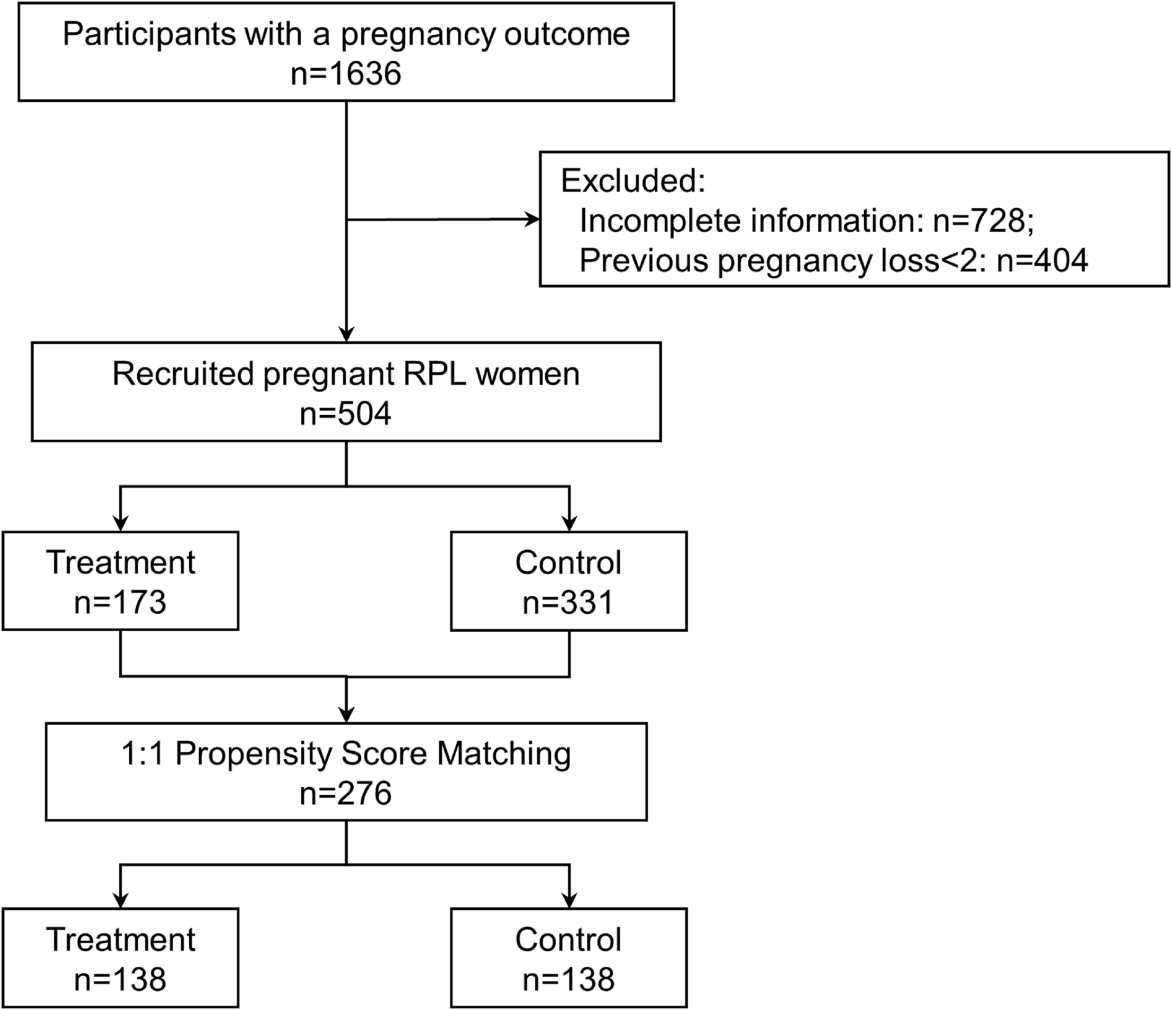
Flow diagram showing the selection process of the treatment and control groups included in the study.

From April 2023 to August 2024, 1,636 pregnant women had a record of delivery or termination of pregnancy in the Lanzhou University Second Hospital. After screening, 504 pregnant RPL women were eventually included, with 173 received IVIG treatment during pregnancy and 331 not received (**Figure 1**). Prior to PSM, there were considerable statistical differences in most variables when comparing the treatment and control groups. Subsequently, we incorporated 276 patients at a 1:1 ratio (Match Tolerance=0.02) following PSM, resulting in a well-balanced distribution of all variables (**Table 1**).

**Table 1:** Characteristics of the study population with or without IVIG use during pregnancy.

Characteristics	Before matching			After matching		
	Treatment (n=173)	Control (n=331)	*P* value	Treatment (n=138)	Control (n=138)	*P* value
Age, years	32.25 ± 4.28	32.29 ± 4.14	0.934	32.46 ± 4.34	32.07 ± 4.20	0.456
Pre-pregnancy BMI, kg/m^2^			0.735			0.976
Underweight (<18.5)	17 (9.8)	29 (8.8)		14 (10.1)	14 (10.1)	
Normal weight (18.5–24.9)	135 (78.0)	254 (76.7)		108 (78.3)	110 (79.7)	
Overweight (25.0−29.9)	20 (11.6)	42 (12.7)		15 (10.9)	14 (10.1)	
Obese (≥30.0)	1 (0.6)	6 (1.8)		1 (0.7)	0 (0.0)	
Age of menarche, years	13.14 ± 1.83	12.98 ± 1.36	0.259	13.16 ± 1.89	12.94 ± 1.24	0.258
Menstrual cycle			0.409			>0.999
Regularity	143 (82.7)	263 (79.5)		111 (80.4)	111 (80.4)	
Irregularity	30 (17.3)	68 (20.5)		27 (19.6)	27 (19.6)	
Educational background			0.026			0.142
With a college/university degree	133 (76.9)	282 (85.2)		103 (74.6)	114 (82.6)	
Without a college/university degree	40 (23.1)	49 (14.8)		35 (25.4)	24 (17.4)	
Number of previous pregnancy losses			0.624			0.250
2	115 (66.5)	212 (64.0)		97 (70.3)	87 (63.0)	
≥3	58 (33.5)	119 (36.0)		41 (29.7)	51 (37.0)	
Type of previous pregnancy losses			0.046			0.769
Primary	168 (97.1)	306 (92.4)		133 (96.4)	131 (94.9)	
Secondary	5 (2.9)	25 (7.6)		5 (3.6)	7 (5.1)	
Number of previous early pregnancy losses			0.343			0.895
0	1 (0.6)	2 (0.6)		1 (0.7)	1 (0.7)	
1	32 (18.5)	83 (25.1)		27 (19.6)	29 (21.0)	
2	62 (35.8)	108 (32.6)		51 (37.0)	49 (35.5)	
3	22 (12.7)	28 (8.5)		15 (10.9)	11 (8.0)	
≥4	3 (1.7)	11 (3.3)		3 (2.2)	6 (4.3)	
Unknown	53 (30.6)	99 (29.9)		41 (29.7)	42 (30.4)	
Number of previous late pregnancy losses or stillbirths			0.078			0.366
0	71 (41.0)	106 (32.0)		56 (40.6)	49 (35.5)	
1	9 (5.2)	30 (9.1)		7 (5.1)	13 (9.4)	
≥2	1 (0.6)	7 (2.1)		1 (0.7)	3 (2.2)	
Unknown	92 (53.2)	188 (56.8)		74 (53.6)	73 (52.9)	
Number of previous biochemical pregnancy losses			0.729			0.848
0	85 (49.1)	165 (49.8)		70 (50.7)	71 (51.4)	
1	58 (33.5)	101 (30.5)		46 (33.3)	42 (30.4)	
≥2	30 (17.3)	65 (19.6)		22 (15.9)	25 (18.1)	
IVF-ET	64 (37.0)	91 (27.5)	0.033	51 (37.0)	44 (31.9)	0.447
Co-medications during pregnancy						
Prednisone	165 (95.4)	160 (48.3)	<0.001	130 (94.2)	132 (95.7)	0.785
Hydroxychloroquine	39 (22.5)	44 (13.3)	0.008	29 (21.0)	25 (18.1)	0.649
Aspirin	76 (43.9)	94 (28.4)	0.001	59 (42.8)	56 (40.6)	0.807
Ciclosporin	39 (22.5)	43 (13.0)	0.007	28 (20.3)	29 (21.0)	>0.999
rhG-CSF	153 (88.4)	146 (44.1)	<0.001	119 (86.2)	116 (84.1)	0.735
TNFi	25 (14.5)	6 (1.8)	<0.001	6 (4.3)	4 (2.9)	0.749

Data are shown as mean ± standard deviation or frequency with percentage. BMI, body mass index; IVF-ET, *In vitro* Fertilization-Embryo Transfer; rhG-CSF, recombinant human granulocyte-colony stimulating factor; TNFi, tumor necrosis factor-α inhibitor.

### 3.1 Pregnancy outcomes

According to **Table 2**, the administration of IVIG during pregnancy was linked to a higher likelihood of LBR when compared to the control group (60.1% vs. 44.9%; adjusted OR [aOR]=1.960, 95%CI 1.183–3.248, *P* < 0.05). When cases with abnormal karyotypes were further excluded, the LBR for the IVIG treatment group remained above that of the control group (64.3% vs. 46.3%; aOR=2.187, 95%CI 1.300–3.679, *P* < 0.05). Our findings maintained consistent even after controlling for various confounding variables. Nevertheless, no significant correlation was found between the use of IVIG during pregnancy and rates of preterm births, birth weights, or neonatal unit admissions.

**Table 2:** Effects of IVIG use on pregnancy outcomes compared with the control group.

Outcomes	Treatment (n=138)	Control (n=138)	Crude OR (95%CI)	*Adjusted OR (95%CI)
Live births	83 (60.1)	62 (44.9)	1.850 (1.147-2.984)	1.960 (1.183-3.248)
Live births excluding abnormal karyotypes	83/129 (64.3)	62/134 (46.3)	2.095 (1.277-3.438)	2.187 (1.300-3.679)
Pregnancy losses	55 (39.9)	76 (55.1)		
Biochemical pregnancy	14 (10.1)	37 (26.8)		
Ectopic pregnancy	0 (0.0)	2 (1.4)		
Preterm births <37 weeks gestation^a^	3/56 (5.4)	2/36 (5.6)	0.962 (0.153-6.061)	0.535 (0.059-4.833)
Birth weights (g)^b^	3158.19 ± 571.23	3177.50 ± 428.71		
Neonatal unit admission^c^	4/55 (7.3)	3/32 (9.4)	0.758 (0.159-3.625)	0.903 (0.127-6.417)

Data are shown as mean ± standard deviation or frequency with percentage.

*Adjusted for maternal age, pre-pregnancy BMI, number of previous pregnancy losses, type of previous pregnancy losses, and co-medications during pregnancy.

a Patients with unknown gestational age at delivery were excluded from the denominator.

b In the treatment group, 56 patients were included; 3 with twin pregnancies were excluded, resulting in 53 patients for this analysis. Birth weight data were available for 36 patients in the control group.

c Patients with unknown neonatal unit admission status were excluded from the denominator.

### 3.2 Stratified analysis

We conducted a stratified analysis of the mean daily dose and gestational weeks of the initial medication of IVIG administration during pregnancy, respectively.

**Figure 2:**
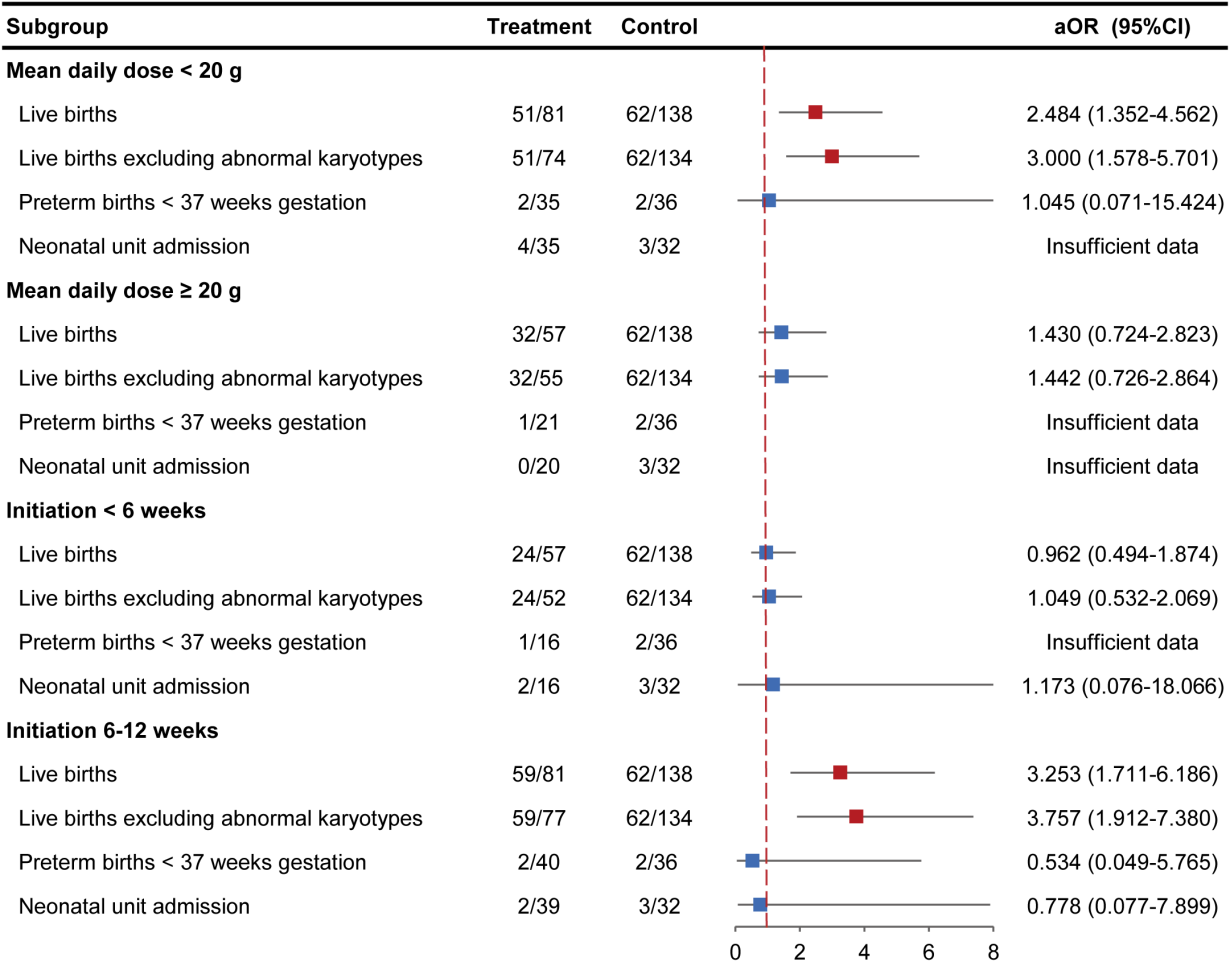
Stratified analysis of mean daily IVIG dose and gestational week at IVIG initiation during pregnancy on live birth rate and other pregnancy outcomes.

The results indicated that taking IVIG less than 20 g/d could increase the odds of LBR (63.0% vs. 44.9%; aOR=2.484, 95%CI 1.352–4.562, *P* < 0.05). This advantage persisted in the IVIG treatment group after excluding cases with abnormal karyotypes (68.9% vs. 46.3%; aOR=3.000, 95%CI 1.578–5.701, *P* < 0.05). In contrast, no significant association was observed between a mean daily dose of IVIG ≥20 g and LBR, whether analyzed in the overall cohort or after excluding cases with abnormal karyotypes. Notably, regardless of the daily dose level, IVIG administration showed no statistically significant effects on preterm birth rate, birth weight, or neonatal unit admission rate (*P*>0.05). (**Figure 2**; **Supplementary Table S1**).

Furthermore, RPL women who commenced IVIG treatment between 6–12 weeks’ gestation exhibited significantly higher LBR in comparison with the control group (72.8% vs. 44.9%; aOR=3.253, 95%CI 1.711–6.186, *P* < 0.05). This benefit was further amplified in the subgroup without abnormal karyotypes (76.6% vs. 46.3%; aOR=3.757, 95%CI 1.912–7.380, *P* < 0.05). However, IVIG administered before 6 weeks’ gestation showed no significant difference in LBR versus controls (42.1% vs. 44.9%; aOR=0.962, 95%CI 0.494–1.874, *P*>0.05). (**Figure 2**; **Supplementary Table S2**).

We further conducted subgroup analyses stratified by conception method (natural conception vs. ART) and tested for interaction. The results showed that IVIG treatment was associated with a significantly higher LBR in the ART group in comparation to the control group (aOR=3.319, 95%CI 1.319–8.350), whereas in the natural conception subgroup, the association was not statistically significant (aOR=1.601, 95%CI 0.821–3.123). However, the interaction between IVIG treatment and conception method did not reach statistical significance (*P* for interaction=0.298). Similar trends were observed when pregnancies with abnormal karyotypes were excluded (*P* for interaction=0.448). (**Supplementary Table S3**).

## 4 Discussion

This research examined the link between the use of IVIG during pregnancy and pregnancy outcomes in patients with RPL. Through the analysis of data involving 276 RPL patients, this retrospective cohort study revealed that IVIG administration during pregnancy correlated with a notably higher LBR when compared to the control group. Furthermore, this positive association remained significant even after cases with abnormal karyotypes were excluded. Subgroup analyses further revealed that IVIG initiated between 6–12 weeks of gestation and at a mean daily dose <20 g was linked to the greatest improvement in LBR. In contrast, earlier initiation (<6 weeks) or higher daily doses (≥20 g) showed no clear benefit. Additionally, IVIG treatment had no significant effect on preterm birth (<37 weeks of gestation), neonatal unit admission, or birth weight. These findings suggest that appropriately timed and dosed IVIG may offer clinical benefit for selected RPL patients, supporting its rational use in practice.

RPL is often associated with dysregulated maternal immune tolerance at the maternal-fetal interface. Proposed immune abnormalities include elevated Th1/Th2 cytokine ratios, increased peripheral or uterine NK cell number/activity, and reduced regulatory T (Treg) cell function (20, 21). IVIG has broad immunomodulatory actions that may correct these imbalances. Importantly, IVIG can suppress NK cell cytotoxicity: it upregulates inhibitory receptors such as CD94 on NK cells, thereby dampening their killing activity (22). Studies in RPL patients have shown that IVIG treatment reduces peripheral NK cell counts and cytotoxic markers, often correlating with pregnancy success (23, 24). IVIG also skews the cytokine milieu toward an anti-inflammatory (Th2) profile by decreasing Th1 cytokines and increasing Th2 cytokines, and such a shift in the Th1/Th2 balance in favor of tolerance (25, 26), which is associated with higher LBR (25).

Our findings provide real-world evidence suggesting that IVIG use during pregnancy may be associated with higher likelihood of live birth in selected patients with RPL. In line with previous observational studies, Mu et al. reported markedly higher LBR in IVIG-treated unexplained RPL patients (77.7% vs. 53.7%, aOR=4.38) (15), and Kim et al. observed a 73.5% LBR in RPL patients receiving repeated IVIG and aspirin (27). Shi et al.’s meta-analysis of 15 studies (902 patients) also concluded that IVIG can significantly increase LBR (OR = 3.06, 95%CI 1.23-7.64) (28). However, high-quality evidence from randomized trials and systematic reviews has been inconsistent (9). A RCT by Christiansen et al. in women with RPL found no significant difference in LBR between IVIG and placebo (54.8% vs. 50.0%, RR = 1.11) (7). Likewise, a recent meta-analysis of RCTs by Ling et al. reported no overall LBR improvement with IVIG (OR = 1.07) (29). A recent meta-analysis by Kofod et al. also did not demonstrate an overall benefit of IVIG on LBR (10). Notably, this analysis suggested that LBR after IVIG increased with the number of previous pregnancy losses, with a potential benefit observed in women with at least six prior losses.

In addition, this study demonstrated that IVIG initiation between 6–12 weeks of gestation and at mean daily doses <20 g was significantly improved LBR, whereas earlier administration (<6 weeks) or higher daily doses (≥20 g) did not demonstrate clear benefit. Differences in patient selection, timing of initiation, and dosing strategies likely contribute to the heterogeneity of reported outcomes. For example, Christiansen et al. enrolled women with secondary RPL and at least four prior losses (7), whereas our cohort included a mixture of primary and secondary losses, with most patients experiencing two prior pregnancy losses.

Similarly, Yamada et al. reported improved outcomes with high-dose IVIG (20 g daily for 5 days) and very early initiation (4–5 weeks) in women with more severe RPL histories (≥4 losses) (30). However, our data suggest that such intensive regimens may not be necessary, or beneficial, for women with fewer prior losses. Furthermore, we conducted a *post-hoc* power analysis based on unadjusted estimates (crude OR). The association between IVIG use initiated at 6–12 weeks and improved LBR (crude OR = 3.287, 95%CI 1.816–5.951) yielded a relatively high statistical power of 0.875, suggesting a robust and reliable effect. In contrast, the association observed for IVIG dose <20 g/d (crude OR = 2.084, 95%CI 1.188–3.656) supported by lower power (0.644), indicating that findings dosage should be interpreted with caution. These considerations highlight the need for further research to clarify dose-response relationships and optimal treatment windows. Besides, our subgroup analysis by conception method indicated a stronger association between IVIG use and live birth in women conceived via ART compared with natural conception. Nevertheless, the interaction test did not reach statistical significance, suggesting that the overall effect of IVIG is not strictly dependent on conception method.

Efforts to reduce pregnancy loss are essential not only to alleviate the emotional and psychological burden on affected couples, but also to decrease healthcare utilization related to RPL, fertility treatments, and associated complications. Although IVIG is widely used in clinical practice for RPL, but evidence-based guidance on its optimal dosing and timing remains limited. To the best of our understanding, this study represents one of the largest real-world investigations into the use of IVIG among pregnant RPL women within a Chinese demographic. By applying PSM, we improved comparability between treatment groups and reduced the influence of measured confounders. Furthermore, we took into account the dose-response relationship of the medication and managed confounding variables associated with treatment indications. Notably, the observed benefit of IVIG on live birth was present even in pregnancies with abnormal karyotypes, suggesting that its therapeutic effect may extend to a broader RPL population.

However, several limitations should be taken into account when interpreting the findings. First, as a retrospective study, the information regarding medication for patients was derived from the medical record database, which could potentially lead to misclassification or underestimation of both the IVIG dose and the duration it was administer during pregnancy. Although PSM was applied to improve comparability between the groups, residual confounding from unmeasured variables cannot be excluded, such as ethnicity, smoking, alcohol, family income, and education. Besides, IVIG therapy, due to its high cost and inconvenient to administer, may have been preferentially prescribed to women perceived by clinicians to have a more favorable prognosis. Additionally, this was a single-center study conducted in a Chinese population, which may limit the generalizability of the findings to other ethnic groups or healthcare settings. Despite these limitations, the present study provides valuable real-world evidence on the use of IVIG in women with RPL and offers insights into the effects of administration timing and dosing.

In conclusion, IVIG use during pregnancy was associated with significantly higher LBR in women with RPL, especially when initiated between 6–12 weeks of gestation at doses <20 g/d. This association remained significant even after excluding cases with abnormal karyotypes. These findings suggest that both the dosage and timing of IVIG administration may influence treatment effectiveness.

## Data Availability

The original contributions presented in the study are included in the article/**Supplementary Material**. Further inquiries can be directed to the corresponding author.
