# Supplementary material for: Early intravenous immunoglobulin use improves live birth outcomes in women with recurrent pregnancy loss: a propensity score–matched cohort study
Source: Front Immunol. 2026 Jan 28;17:1689166. doi: 10.3389/fimmu.2026.1689166 (PMC12891192; doi:10.3389/fimmu.2026.1689166)
Supplement: Supplementary file 1 [file Table1.docx]

**Supplementary Table 1.** Effects of mean daily IVIG dose on pregnancy outcomes compared with the control group

| **Outcomes** | **Control (n=138)** | **Treatment**  **(<20 g/d) (n=81)** | **Crude OR(95%CI)** | ***Adjusted OR(95%CI)** | **Treatment**  **(≥20 g/d) (n=57)** | **Crude OR(95%CI)** | ***Adjusted OR(95%CI)** |
| --- | --- | --- | --- | --- | --- | --- | --- |
| Live births | 62 (44.9) | 51 (63.0) | 2.084(1.188-3.656) | 2.484(1.352-4.562) | 32 (56.14) | 1.569(0.843-2.921) | 1.430(0.724-2.823) |
| Live births excluding abnormal karyotypes | 62/134 (46.3) | 51/74 (68.9) | 2.575(1.416-4.683) | 3.000(1.578-5.701) | 32/55 (58.2) | 1.616(0.857-3.047) | 1.442(0.726-2.864) |
| Pregnancy losses | 76 (55.1) | 30 (37.0) |  |  | 25 (43.86) |  |  |
| Biochemical pregnancy | 37 (48.7) | 9 (11.1) |  |  | 5 (8.8) |  |  |
| Ectopic pregnancy | 2 (2.6) | 0 (0.0) |  |  | 0 (0.0) |  |  |
| Preterm births <37 weeks gestation^a^ | 2/36 (5.6) | 2/35 (5.7) | 1.030(0.137-7.748) | 1.045(0.071-15.424) | 1/21 (4.8) | 0.850(0.072-9.981) | - |
| Birth weights (g)^b^ | 3177.50±428.71 | 3207.27±563.84 |  |  | 3148.75±596.11 |  |  |
| Neonatal unit admission^c^ | 3/32 (9.4) | 4/35 (11.4) | 1.247(0.257-6.057) | - | 0/20 (0.0) | - | - |

**Note:** Data are shown as mean±standard deviation or frequency with percentage.

*Adjusted for maternal age, pre-pregnancy BMI, number of previous pregnancy losses, type of previous pregnancy losses, and co-medications during pregnancy.

^a^ Patients with unknown gestational age at delivery were excluded from the denominator.

^b^ In the treatment group, 56 patients were included; 3 with twin pregnancies were excluded, resulting in 53 patients for this analysis. Birth weight data were available for 36 patients in the control group.

^c^ Patients with unknown neonatal unit admission status were excluded from the denominator.

**Supplementary Table 2.** Effects of gestational age at initiation of IVIG on pregnancy outcomes compared with the control group

| **Outcomes** | **Control (n=138)** | **Treatment**  **(Initiation <6 weeks) (n=57)** | **Crude OR(95%CI)** | ***Adjusted OR(95%CI)** | **Treatment**  **(Initiation 6-12 weeks) (n=81)** | **Crude OR(95%CI)** | ***Adjusted OR(95%CI)** |
| --- | --- | --- | --- | --- | --- | --- | --- |
| Live births | 62 (44.9) | 24 (42.1) | 0.891(0.478-1.663) | 0.962(0.494-1.874) | 59 (72.8) | 3.287(1.816-5.951) | 3.253(1.711-6.186) |
| Live births excluding abnormal karyotypes | 62/134 (46.3) | 24/52 (46.2) | 0.995(0.524-1.892) | 1.049(0.532-2.069) | 59/77 (76.6) | 3.806(2.032-7.130) | 3.757(1.912-7.380) |
| Pregnancy losses | 76 (55.1) | 33 (57.9) |  |  | 22 (27.2) |  |  |
| Biochemical pregnancy | 37 (48.7) | 12 (21.1) |  |  | 2 (2.5) |  |  |
| Ectopic pregnancy | 2 (2.6) | 0 (0.0) |  |  | 0 (0.0) |  |  |
| Preterm births <37 weeks gestation^a^ | 2/36 (5.6) | 1/16 (6.3) | 1.133(0.095-13.481) | - | 2/40 (5.0) | 0.895(0.119-6.703) | 0.534(0.049-5.765) |
| Birth weights (g)^b^ | 3177.50±428.71 | 3386.00±552.30 |  |  | 3105.92±566.01 |  |  |
| Neonatal unit admission^c^ | 3/32 (9.4) | 2/16 (12.5) | 1.381(0.207-9.228) | 1.173(0.076-18.066) | 2/39 (5.13) | 0.523(0.082-3.337) | 0.778(0.077-7.899) |

**Note:** Data are shown as mean±standard deviation or frequency with percentage.

*Adjusted for maternal age, pre-pregnancy BMI, number of previous pregnancy losses, type of previous pregnancy losses, and co-medications during pregnancy.

^a^ Patients with unknown gestational age at delivery were excluded from the denominator.

^b^ In the treatment group, 56 patients were included; 3 with twin pregnancies were excluded, resulting in 53 patients for this analysis. Birth weight data were available for 36 patients in the control group.

^c^ Patients with unknown neonatal unit admission status were excluded from the denominator.

**Supplementary Table 3.** Subgroup analysis of live birth rate by conception method in women with RPL treated with IVIG

| **Subgroup** | **Treatment** | **Control** | **Crude OR(95%CI)** | ***P* for interaction** | ***Adjusted OR(95%CI)** | ***P* for interaction** |
| --- | --- | --- | --- | --- | --- | --- |
| Live births |  |  |  | 0.172 |  | 0.298 |
| ART | 30/51 (58.8) | 14/44 (31.8) | 3.061(1.315-7.124) |  | 3.319(1.319-8.350) |  |
| Natural conception | 53/87 (60.9) | 48/94 (51.1) | 1.494(0.827-2.697) |  | 1.601(0.821-3.123) |  |
| Pregnancy loss excluding abnormal karyotypes |  |  |  | 0.298 |  | 0.448 |
| ART | 30/49 (61.2) | 14/42 (33.3) | 3.158(1.335-7.472) |  | 3.628(1.389-9.479) |  |
| Natural conception | 53/80 (66.3) | 48/92 (52.2) | 1.799(0.970-3.339) |  | 1.882(0.936-3.785) |  |

**Note:** Data are shown as frequency with percentage.

*Adjusted for maternal age, pre-pregnancy BMI, number and type of previous pregnancy losses, and co-medications during pregnancy.
